# Integrative medicine and human health - the role of pre-, pro- and synbiotics

**DOI:** 10.1186/2001-1326-1-6

**Published:** 2012-05-28

**Authors:** Stig Bengmark

**Affiliations:** 1 Division of Surgery & Interventional Science, University College London, 4th floor, 74 Huntley Street, London, WC1E 6AU, UK; 2 185 Barrier Point Rd, London, E162SE, UK

## Abstract

Western lifestyle is associated with a sustained low grade increase in inflammation -increased levels of endotoxin in the body and increased activation of Toll-like receptors and neutrophils, which leads to impaired immunity and reduced resistance to disease, changes which might explain the epidemic of chronic diseases spreading around the globe. The immune system cannot function properly without access to bacteria and raw plants, rich not only in bacteria but also in plant fibre, antioxidants, healthy fats and numerous other nutrients. Modern food technology with plant breeding, separation, condensation of food ingredients, heating, freezing, drying, irradiation, microwaving, are effective tool to counteract optimal immune function, and suspected to be a leading cause of so called Western diseases. Supply of pre-, pro-, and synbiotics have sometimes proved to be effective tools to counteract, especially acute diseases, but have often failed, especially in chronic diseases. Thousands of factors contribute to unhealth and numerous alterations in life style and food habits are often needed, in order to prevent and cure “treatment-resistant” chronic diseases. Such alterations include avoiding processed foods rich in pro-inflammatory molecules, but also a focus on consuming substantial amounts of foods with documented anti-inflammatory effects, often raw and fresh green vegetables and tubers such as turmeric/curcumin.

## Review

### Introduction

Human life without access to plants and bacteria would be miserable. Plants and bacteria, which have existed for billions of years, have often robust protection system, which can be used by humans. Our Palaeolithic forefathers did on annual basis receive their daily food from at least five hundred plants and also, as the food they ate often were stored in the soil, a rich supply of various microorganisms. The food of modern food is based on nutrients received from only a small number of plants; 80% of the nutrients come from 17 plants and 50% of the calories from eight grains. Furthermore, the main part of Western foods is extensively processed; growth enhancement, separation, condensing, drying, freezing, irradiation, burning, microwaving, toasting, adding various ingredients and especially heating. It is well-known that some important plant ingredients start disappearing already when heated above 28^o^C, important plant enzymes and microbes above 42^o^ C, dys-functioning proteins be added above 80^o^C and heterocyclic amines and also trans-fatty acids from about 130^o^C, and increasing as the heating of the food increases further, all changes being negative to human health.

Among the dys-functioning proteins produced during heating of foods are the so called Maillard products, often referred to as advanced glycation and advanced lipoxidation end products, and abbreviated as AGEs and ALEs. Among foods rich in AGEs and ALEs are: dairy products especially powder milk (frequently used in enteral nutrition and baby formulas, as well as in numerous foods such as ice cream), cheese, bakery products (bread crusts, crisp breads, pretzels, biscotti) and cereals (rice crispies), overheated (especially deep-fried and oven-fried) meat and poultry but also fish, drinks like coffee and coca cola, Asian sauces including Chinese soy sause, balsamico products and smoked foods in general - for further information, see Goldberg et al [1,2]. The consumption of such foods, often main constituents in fast foods, have increased dramatically in recent decades, much in parallel to the endemic of chronic diseases.

### Deranged and dys-functioning immune system

Numerous chemical substances, additives to foods and pharmaceutical drugs, seem to derange the immune system. It is clear, even if not fully investigated, that a large number of chemicals, when consumed, have a strong negative influence on the immune system and the body’s resistance to disease. In the past, priority was not given to investigate eventual negative effects on the innate immune systems of consumed food additives and pharmaceutical drugs. It has long been known that antibiotics suppress various immune functions, and especially macrophage activities such as chemiluminescence response, chemotactic motility, bactericidal and cytostatic ability and similar negative effects have also been seen with other commonly used drugs such as H_2_-blockers, proton inhibitors and surface-protection agents.

Several other factors increase the degree of systemic inflammation in the body:

*Impaired hormonal homeostasis* increases oxidative stress/ release of free radicals, intracellular accumulation of “waste products”, inhibits apoptosis, disturbs repair mechanisms, reduces gene polymorphism, increases premature shortening of telomeres and reduces immune defence and resistance to disease, changes often observed in premature aging and in various several chronic diseases [3].

*Low level in the body of vitamin D* and subsequent secondary hyperparathyroidism [4].

*Low levels in the body of antioxidants* such as folic acid and glutathione and increased levels of homocysteine [5].

*High levels in the body of estrogens*, especially 17β-estradiol, often induced by high consumption of hormone-rich dairy products [6].

*High levels of angiotensin/renin*[7]

*Larger intake of glutenoids*[8].

The reason why attempts to reduce inflammation with the use of probiotics have in the past sometimes failed might be that the pro-inflammatory pressure induced by the underlying disease is simply too high, but also influenced by larger consumption of proinflammatory foods and prescription drugs, many with strong inflammation-enhancing abilities. It is likely that in certain conditions additional measures are needed in order to achieve successful treatment with probiotics. Measures such as reduced supply of proinflammatory foods, restriction in use of pharmaceuticals and a significant increase in intake of such plant foods known to be rich in anti-inflammatory vitamins and antioxidants, especially might various polyphenols be needed - see further below.

### Effects of plant fibres on systemic inflammation

It should be observed that various seeds, nuts, beans and peas are especially rich in fibre, foods which no longer are eaten in the quantities they deserve. A common recommendation of minimum daily fibre intake is in the range of, 30–35 g per day, which roughly corresponds to about half a kg of fruits and vegetables, or, as often expressed, 5 to 8 fresh fruits and vegetables per day. The recommendations for children above the age of 2 y are usually defined as age + 5 g/day. No precise recommendation exists yet about intake of fibre under different conditions of disease. The daily intake of dietary fibre is unsatisfactory in all Western countries, especially among people with low level of education and low income. In the US for example, the estimated daily intake of fibre is app 14–15 g/day or about 50% of what is recommended, and far below the 60–80 g/d of substrate required to maintain a large bowel flora of 10^14^ microorganisms, known to be typical for a healthy and well-functioning human colon. Most Americans and Europeans have lost the ability to maintain a large proportion of what can be regarded as a natural flora [9]. A study in a North-European population found *Lactobacillus plantarum, Lactobacillus rhamnosus* and *Lactobacillus paracasei* ssp *paracasei* on the rectal mucosa of healthy humans only in 52%, 26% and 17% respectively [10]. The colonization rate with other, commonly milk-born probiotic bacteria, such *Lactobacillus casei, Lactobacillus reuteri* and *Lactobacillus acidophilus* was in the same study only 2%, 2% and 0% respectively.

Commonly consumed cooked roots and other starchy vegetables, grains, consumed as bread, cereals and porridge, but also most of the fruits, consumed in Western countries, contain relatively little fibre, usually no more than 1–3 g/serving. The largest amount of consumed plant fibre is provided by resistant starch (rich in raw tubers such as potato and in unripe green banana). Cooked tubers, especially potatoes will re-crystallize when allowed to cool after cooking. The daily consumption of this type of fibre varies from one individual to another with several hundred per cent (app 8–40 g/d). The second largest source of fibre is non-starch polysaccharides (app 8–18 g/d). The third group of fibre is oligosaccharides (onions, artichoke, banana, cecoria), which although important to health, regrettably today is consumed in much too small quantities (app 2–8 g/d) [11].

### Function of dietary fibres

Supplemented fibres are associated with several health benefits. The best documented physiological effects, in addition to providing energy and nutrients to the host and flora, are that they:

· change in mucosal structure, increase mucosal growth and improve mucosal function.

· increase in intestinal flora, relieve constipation, reduce production of putrifactive gases and provide resistance to invading microorganisms

· reduce serum triglycerides, serum cholesterol and VLD lipoproteins

· reduce the glycemic response to eating

· improve water and electrolyte balance and increase bioavailability and absorption of minerals such as Ca, Mg Fe and Zn.

Consumption of medical fibres should always be regarded as a surrogate for not consuming enough of fresh fruits and vegetables. Generally processed fibers are much less efficient in the body compared to natural fibres. There is no solid information to support that supplementation of medical fibres to healthy individuals eating a diet rich in fruits and vegetables is associated with additional health benefits. Medical fibres are mainly needed because the individual has lost the ability to consume enough of fresh fruits and vegetables. This is often the situation in persons with severe allergy, in old and debilitated persons and in persons with some GI disorders, such as short bowel syndrome and advanced diverticular disease. This is also most often the condition for critically ill patients, for whom enteral supply of concentrates of medical fibres has become a most valuable clinical tool. It must, however, always be remembered that bioactive fibres during the processing have lost their content of numerous important antioxidants and nutrients, some of which when possible should be separately supplemented, and whenever possible complemented by supply of fresh fruits and vegetables.

### Health benefits of increased plant fibre consumption

Significant information on beneficial effects from increased intake of plant fibres and so called prebiotics exists mainly for two large groups of diseases: 

*Blood glucose control/prevention of type 2 diabetes.* Fibre is a slow release system for delivery of glucose to the body. Fibre, regularly supplied to patients with diabetes will significantly reduce the level of blood glucose and the need of insulin. Studies suggest that the most pronounced effects of fibres on glycemic index are obtained by water-soluble fibres. Guar-gum is in this respect by far the most clinically tried fibre and will, as based on 15 different studies, reduce blood glucose to almost half (44%).

*Lipid control/prevention of coronary heart disease.* Soluble fibres such as pectin, guar gum, betaglucans (oat) reduce significantly blood cholesterol both in hypercholesterolemic and normocholesterolemic individuals, effects not found when non-soluble fibres such as cellulose and wheat bran are tried. Soluble fibres are excellent substrates for production in the large intestine of short chain fatty acids (SCFAs), known prevent leaky gut and also to reduce the levels of cholesterol/s.

A meta-analysis reports statistically significant protective effects against coronary heart disease in 14/16 studies [12]. In addition, larger fibre consumption is also known to reduce clotting and increase fibrinolysis, also important for prevention of arterial wall plaques and of thrombosis formation.

### Clinical use of fibres

Substances, important to health: amino acids such as arginine, glutamine, histidine, taurine, various sulphur and related amino acids, polyamines, omega-fatty acids, numerous vitamins and antioxidants are all to a great extent supplied to the body from plants. One cannot expect any significant amount of antioxidants to be delivered to the lower level of the gastrointestinal tract, if not carried by plant fibres. It is important to remember that key nutrients such as omega-3 fatty acids, glutamine, glutathione and several other nutrients are heat-sensitive and do not tolerate processing or storage to any larger extent. Plant fibres, which have been dried, heated up or micro-waved cannot be expected to contain any larger amounts of these, with this raw and unprocessed foods are needed. It is highly desirable that, whenever possible, the supply of commercial nutrition formulas are complemented by supply of fresh fruit and vegetable juices, as locally produced as ever possible. It is also desirable that several different fibres are supplied in parallel, and that both soluble and non-soluble fibres are supplied. For example, oat fibres are mainly metabolized in the proximal colon, while wheat fibres are known to be effective in the distal part of the colon, e.g. the part of colon where most cancers are localized. Oat has mainly shown sepsis-reducing effects while wheat has mainly been effective in cancer prevention.

Among the fibres commonly used in clinical nutrition are:

*Algal fibres.* Most of the algal fibres are resistant to hydrolysis by human endogenous digestive enzymes, but are to various degrees fermented by colonic flora. The soluble fibres consists in lamarans (a sort of β-glucan associated with mannitol residues), fucans (sulphated polymers associated with xylose, galactose and glucoronic acid) and alginates (mannuronic and guluronic acid polymers). The insoluble algal polymers consist mainly in cellulose. Fermentation of alginates yields high levels of acetate (80%), while lamirans yield preferably butyrate (16%). It is most likely that algal fibres will within a few years be routinely used in clinical nutrition.

*Fructans.* Fructans starches and sucrose serve in the plant as its energy reserve. These substances are also produced by bacteria and fungi. Fructans are said to enhance the tolerance of the plant to stressful conditions and make it possible for the plants to survive under harsh conditions, such as low temperature and draft. The most well-known fructans are inulin (rich in chicory, artichoke, onions, and banana) and phleins (rich in various grasses). This far it is mainly inulin, which has been tried in human nutrition. Various oligosaccharides are reported to stimulate the flora and especially the growth of *Lactobacilli* and *Bifidobacteria* in the large intestine and to reduce the content of potentially pathogenic microorganisms (PPMs) in the intestine. Increase in the *Bifidobacteria* flora is regarded as especially favourable as *Bifidobacteria* are known to produce important vitamins, among them thiamine, folic acid, nicotinic acid, pyridoxine and vitamin B_12_, which is of great importance for health. A fructan called neokestose, found in onion, is reported to have even better ability than inulin to promote growth of LAB. Also supplementation of fructans is reported to reduce concentrations in serum of insulin, cholesterol and triacylglycerol. It is also reported to promote absorption of calcium and other minerals. Other oligosaccharides such as those extracted from peas and beans especially soya bean oligosaccharide (raffinose and stachyose) and pyrodextrin, produced by pyrolysis of maize and potato starch are also reported to be beneficial for human health.

*Glycomannans.* Glycomannan, a glucose/mannose polymer derived from a plant called *Amorphophallus konjak*, which has several English names such as devil tongue, elephant yam and umbrella arum. It has unique hydroscopic abilities and will on contact with water swell and form a viscous gel, which like other gels will delay gastric emptying and intestinal transit time. It has been shown to be effective in delaying absorption of digestible energy. It has this far mainly been used in Japan and other Asian countries to treat diabetes, hypertension and hypercholesterolemia. Dietary supply of konjak mannans has been shown in experimental animals to alter the flora and reduce tumorigenesis. It is also effective to control of diarrhoea in enteral nutrition, especially in elderly patients, and to increase the *Bifidobacteria* flora.

*Oat gum.* Oat contains a series of interesting compounds, which is the reason why an increasing part the world production of oat goes to the pharmaceutical and cosmetic industries. The amino acid pattern of oat is rather similar to that of human muscle (only that of buckwheat is more alike), and can thus be expected to deliver most of the amino acids needed to build muscles. Oat is rich in water-soluble fibers, β-glucans, and known for their antiseptic properties. Oat is also rich in natural antioxidants, particularly ferulic acid, caffeic acid, hydrocinnamic acid, and tocopherols and oat was before synthetic antioxidants were available extensively used to preserve foods: milk, milk powder, butter, ice-cream, fish, bacon, sausages and other food products sensitive to fat oxidation. Another ingredient richly available in oat is inositol hexaphosphate (phytic acid), a strong antioxidant, particularly known to enhance natural killer cell activity and to suppress tumour growth. Oat is also rich in polyunsaturated fats/polar lipids such as phosphatidylcholine, known for its protective effects of mucosal and cellular surfaces.

*Pectin.* Also pectin is an interesting fibre, extensively used by pharmaceutical and food industry. It has a unique ability to form gels and is commonly used as a carrier of pharmacologically active substances and is common in baby foods. An important finding is that pectin a very strong antioxidant against the three most dominating oxidation damages induced by peroxyl-, superoxide- and hydroxyl radicals. These effects might explain why pectin has the capacity to stimulate the gut-associated immune system and to prevent disruption of the intestinal microflora. Pectins have shown strong protective and healing effects on gastric but also intestinal mucosa, in experimental studies not inferior to what is observed with H_2_-blockers, proton inhibitors and surface-protection agents [13,14]. Pectin builds a protection layer in the stomach and facilitates maintenance of gastric acidity, important for prevention of colonization of the stomach by pathogens. Pectin is also an excellent substrate for microbial fermentation.

### Lactic acid bacteria key to fermentation of fibres

Not all fibres are easily fermented in the gut. Among the more fermentation-resistant fibres are wheat fibres which usually are not digested until they reach descending colon. Also oligofructans (inulin or phleins) are difficult to ferment and only a small minority of lactic acid bacteria (LAB) are able to do so. When the ability of 712 different LAB to ferment oligofructans was studied, only 16/712 were able to ferment the phleins and 8/712 inulin [15]. Apart from *Lactobacillus plantarum* only three other LAB species, *Lactobacillus paracasei* subsp. p*aracaseiLactobacillus brevis* and *Pediococcus pentosaceus* were able to ferment such semi-resistant fibres. Another study investigated the ability of 28 different LAB to ferment pure fructooligosacharides (FOS). All *Lb plantarumLb casei* and *Lb acidophilus* strains studied and most *Bifidobacteria* metabolized FOS, in contrast to yoghurt bacteria such as *Lb bulgaricus* and *Streptococcus thermophilus* and also *Lactobacillus strain GG,* which all were unable to ferment these fibres [16].

### Clinical experience with supplemented plant fibres

#### Plant fibres in constipation

Chronic constipation is one of the most common disorders in Western countries. Its etiology remains despite numerous clinical, pathophysiologic, and epidemiologic studies unclear, but it is suggested high intake of dairy products and intake of plant fibres play a significant role in its pathogenesis. A randomized sample of 291 children with idiopathic chronic constipation was in a case control study compared with 1602 healthy controls [17]. Constipation was clearly negatively correlated with low intake of cellulose and pentose fibres (*p* < 0.001). Fructo-oligosaccharides (FOS) may also have potential benefits in constipation, since they exhibit many soluble dietary fibre-like properties. In a study a total of fifty-six healthy infants, age 16–46 weeks (mean age 32 weeks) were randomly assigned to receive either 0.75 g FOS or placebo added to a serving of cereals for 28 d [18]. The mean number of stools per infant was 1.99 ± 0.62 per day in the FOS-supplemented group compared with 1.58 ± 0.66 in the control group (*P* = 0.02).

#### Plant fibre to prevent and treat diarrhoea

In a large randomized study in acutely ill medical and surgical patients, all requiring enteral nutrition for a minimum of 5 days supplementation of hydrolyzed guar gum was compared to a fibre-free enteral nutrition, the incidence of diarrhoea being 9% with fibre-supplementation, compared to 32% with fibre-free nutrition (p > 0.05) [19]. One of the effects of certain fibres is that it increases the bioavailability and absorption of zinc, which is especially shown for oligosaccharides. Zink supplementation was in a randomized study in 3–59 months children in Bangladesh proven effective to lower both the incidence of diarrhoea and the duration of diarrhea [20]. In another study from Bangladesh 250 g/L of green (unripe) banana (eqv. to two fruits) or 2 g pectin/kg food was compared to a supplemented rice diet in children suffering from persistent diarrhoea [21]. The amounts of and frequency of stools, the duration of diarrhoea, numbers of vomiting, use of oral rehydration and amounts iv fluid solutions given were all significantly better reduced in the the group supplemented green banana and pure pectin. Recovery on third day was seen in 59% in the green banana group, in 55% in the pectin group compared to 15% in the rice only control group.

#### Plant fibre to support mineral absorption

It is well accepted that nutrition is of great importance also for bone health. Most interest has this far focused on calcium and vitamin D. Much less interest has been paid to other important nutrients such as protein, and especially to minerals such as phosphorus, potassium, magnesium and vitamins such as C and K. Recent studies suggests that increased intake of plant fibers, fruits and vegetables is associated with an increased bone mineral density also in elderly subjects, both women and men [22,23]. Of all the many pure fibres available it is mainly the effects of oligosaccharides have been more thoroughly studied, but so far mainly in experimental animals Calcium absorption, bone calcium content, bone mineral density, bone balance and bone formation/bone absorption index are generally reported to be significantly increased, and that as early already as after three weeks of supplementation of a mixture of inulin and fructooligosaccharides.

#### Plant fibre to control weight

No major effects on body weight by supplementation of only prebiotic fibre have this far been reported. The effects of dietary fibre on subjective hunger ratings and weight losses were studied some twenty years ago in members of a weight loss club. One hundred and eight of 135 members completed the trial: 23 controls, 45 on ispaghula granulate and 40 on bran sachets [24]. Both fibre preparations reduced hunger at all meals. The mean (±SD) weight reductions during the trial were 4.6 ± 2.7 kg for the controls, 4.2 ± 3.2 kg for the ispaghula group and 4.6 ± 2.3 kg for the bran group (*p* > 0.05 for both groups). Although supply of dietary fibre immediately before meals did reduce the feeling of hunger did it not provide any additional benefits to the weight reduction. A more recent crossover study compared the effect on satiety of supplementation of 27 ± 0.6 g/d of fermentable fibers (pectin, betaglucan) with similar amounts of non-fermentable fibre (methylcellulose). The daily satiety was significantly increased with non-fermentable (methylcellulose) than with fermentable fibres (betaglucan, pectin) (*p* = 0.01) but no differences were observed in daily energy intake or loss of body weight or body fat [25].

### Plant fibre in inflammatory bowel diseases (IBD)

Although both patients with IBD and irritable bowel syndrome (IBS) are known to under-consume dietary fibres, there is little evidence that lack of dietary fibre plays a role in the pathogenesis of these diseases. The ability of maintaining remission in UC patients by a daily supply of 10 g of Plantago ovata seeds (also called psyllium or Ispaghula husk) was compared with daily treatment with 500 mg of mesalamine and a combination of the two [26] Twelve months of treatment failed to demonstrate any difference in clinical benefits between the three groups. Germinated barley foodstuff (GBF), a by-product from breweries, rich in hemi-cellulose and in glutamine, was tried in 39 patients with mild-to-moderate active UC [27]. Daily supply of 30 g reduced significantly the disease activity, increased concentration of SCFAs, and increased in stool the numbers of *Bifidobacterium and Eubacterium*. It can well be that the observed effect were more due to increased supply of glutamine and other antioxidants such as various B vitamins than to the fibre per se as these compounds are known to be rich in by-products from breweries. Glutamine, as well as other antioxidants, are known to attenuate pro-inflammatory cytokines such as TNF-α and to enhance release of heat shock proteins (HSP-72) [28].

A controlled study using oat bran as fibre source was reported in a study of 22 patients and 10 controls, all with quiescent UC. Daily supply during three months of as much as 60 g of oat bran (eqv to 20 g dietary fibre) resulted in a significant increase in faecal butyrate (average 36%), but also in significant reduction in abdominal pain. All the treated patients tolerated well the large dose of fibre and signs of relapse of disease were seen in none of the colitis patients [29]. Butyrate has been shown to inhibit NF-kB activation of lamina propria macrophages, and to reduce the number of neutrophils in crypts and surface epithelia, as well as the density of lamina propria lymphocytes/plasma cells in patients with ulcerative colitis [30] - findings correlating well with the observed decreased disease activity. Twenty patients with ileal pouch-anal anastomosis received daily for two weeks 24 g of inulin. Significant reduction in inflammation was observed at endoscopy and histology. In addition significant increase in faecal concentrations of butyrate and reductions in faecal pH, faecal content of secondary bile acids, and growth of *Bacteroides fragilis* was observed [31].

#### Plant fibre in irritable bowel disease

Dys-motility disorders are increasingly common in Western Societies. Some evidence suggest that various dys-motility disorders; gastroesophageal reflux problems, infant colic and constipation are all food-related features, and often due to intolerance to cow’s milk proteins [32]. Irritable bowel syndrome (IBS), is a clinical diagnosis based on the occurrence of abdominal distension, abdominal cramps, often increased transit time, more frequent stools, and relief of pain on defecation. The prevalence of the syndrome varies between and 7 and 22%, making IBS the most common functional gastro-intestinal disorder [33]. Unfortunately, no effective pharmaceutical treatment exists, or when existing, is unacceptably toxic [34]. This has resulted in a need for additional modalities for the treatment of IBS. Pre- and probiotics appear in this perspective as attractive alternatives, see further reviews [35,36] especially as early data from human intervention studies but also results from experimental studies in animals clearly demonstrate an impact on the immune system: immune cells of the GALT including Peyer’s patches [37]. However, a consequence of feeding the currently favoured prebiotics (inulin, fructo-oligosaccharides (FOS), trans-GOSs and lactulose,) is increased gas production in the gut, which might preclude prebiotic use where bloating or gas are prominent symptoms such as in diarrhoea-predominant IBS, but might allow their mild laxative properties to be useful in constipation-predominant IBS [38]. Over the years some small open trials have been performed, but this far, no larger and randomized trial has been reported. However, a small open label trial with supplementation of 15 g/day of a mixture of oligofructose (70%) and inulin (30%) reported significant reduction in disease activity (Harvey Bradshaw index fell from 9.8, SD 3.1 to 6.9 SD 3.4, *p* = 0.01) in parallel to a significant increase in faecal bifidobacteria concentration (from 8.8, SD 0.9 log 10 to 9.4, SD 0.9 log10 cells/g dry feces *p* = 0.001). Also the IL-10 positive dendritic cells increased (from 30 to 53% *p* = 0.06), and the percentage of dendritic cells expressing TLR2 and TLR4 increased from 1.7% to 36.8% *p* = 0.08, and from 3.6% to 75.4 *p* = 0.001) [38], respectively which offers hope for the future.

#### Plant fibre in abdominal pain

Other dietary fibres have also been tried in various groups of abdominal pain. A recent Cochrane review was unable to find any evidence that fibre supplements, lactose free diets or lactobacillus supplementation are effective in the management of children with recurrent abdominal pain [39]. However, a study in adult patients reports significant success with other fibres than classical prebiotics. 188 adult IBS patients were classified as having diarrhoea-predominant, constipation-predominant, or changeable bowel habits type IBS and randomly assigned to groups receiving 30 g/day of wheat bran or 5 g/day of guar gum (PHGG [40]. After four weeks, patients were allowed to switch group, depending on their subjective evaluation of their symptoms. Both fibre and PHGG were effective in improving pain and bowel habits. Significantly more patients switched from fibre to PHGG (49.9%) than from PHGG to fibre (10.9%) at four weeks. Intention-to-treat analysis showed a significantly greater success in the PHGG group (60%) than in the fibre group (40%). In addition, significantly more patients in the PHGG group reported a greater subjective improvement than those in the fibre group. It was concluded that improvements in core IBS symptoms were observed with both bran and PHGG, but the latter was better tolerated and preferred by patients [40].

*Plant fibre to control infections .* In an effort to prevent nosocomial pneumonia and sepsis, patients with severe multiple trauma were treated with beta 1–3 polyglucose (glucan) - a component of cell walls of plants and microbes [41]. Pneumonia occurred in 2/21 glucan-treated and in 11/20 patients in the control group (*p* < 0.01). Infectious complications (pneumonia and/or general sepsis) occured in 14% of glucan-supplemented patients versus 65% in the control group (*p* < 0.001). Another study compared the effects of a high-protein formula enriched with fiber but also arginine, and antioxidants with a standard high-protein formula in early enteral nutrition in critically ill patients [42]. The supplemented group had, in comparison to non-supplemented controls a lower incidence of catheter-related sepsis (0.4 episodes/1000 ICU days) than the control group (5.5 episodes/1000 ICU days) ( *p* < .001), but no differences were observed between the groups in incidence of ventilator-associated pneumonia, surgical infection, bacteremia, urinary tract infections, mortality and in long-term survival [42].

### Pre- pro- and synbiotics improve innate immunity

Several plant fibres (prebiotics) and a few LABs (probiotics) have documented significant effects to improve both the function of the innate immune system, the physical barrier, and increase resistance to disease. The hope is that combined supply of these components shall have synergistic, e.g. more than additive effects in boosting the immune system and enforcing the barrier functions. Products which combine pre-and probiotics are called synbiotics and treatments using the combination called synbiotic treatment.

### Choice strains for probiotic use

The choice of pre- and probiotics must be based on scientific evidence. Stronger bioactivities cannot be expected from LAB such as yogurt bacteria, chosen mainly for their palatability. The strains to use must be selected with care and based on extensive preclinical.

It is important to remember that the majority of LABs have no or much limited effects on immune functions and outcome. Constructing synbiotic formulations is especially demanding as most of the LAB used by industry have no or limited ability to ferment bioactive fibres such as inulin or phlein [15], no ability to adhere to human mucus, low antioxidant capacity and most important, do not survive the acidity of stomach and bile acid content.

It has been observed that strains that carry the same name have different and sometimes opposing functions. When the ability of 46 different *Lactococcus lactis* strains to induce production of the cytokines interleukin (IL)-6, IL- 12 and tumor necrosis factor (TNF)-α was studied, it was clearly demonstrated that the extent of induction of IL-6, IL-12 and TNF-α was cearly shown to be strain-specific and not at all related to subspecies, bio-variety, or the source of the isolate. The production of IL-6 varied between 138 and 0 ng/ml IL-12 between 3 and 0 ng/ml TNF-α between 20 and 0 ng/ml between various strains carrying the same name [43].

Unfortunately few studies have looked at the synergistic effects of simultaneous supply of LAB and fibres - synbiotics. Natural foods supplies both LAB and a great variety of plant fibres. Combination of several fibres has been shown to lead to additive effects on microbial ecosystem and immune responses [44], and multi-species probiotics documented to be superior to single-species probiotics, showing increased ability to enhance growth, reduce antibiotic associated diarrhoea, prevent infections (*S typhimurium*) and reduce pathogenic colonisation (*E coli*) [45] Although some studies have used various synbiotic compositions, only two such compositions have been produced after extensive preclinical studies:

1. *An unistrain/unifibre composition,* produced (Probi AB, Lund Sweden) by fermentation of oat meal with *L. plantarum* strain 299, containing 10^9^ of LAB and app 10 g oat fibre [46]. In a few studies a commercial fruit juice, PRO VIVA™ containing 10^7^ of a related *L. plantarum* strain called 299 V, (Skånemejerier, Malmö, Sweden) is also tried.

2. *A multistrain/multifibre composition,* called Synbiotic 2000™, consisting in a mixture of 10^10^ and a Synbiotic FORTE™ with 10^11^ of each of four LAB: *Pediacoccus pentosaceus* 5–33:3, *Leuconostoc mesenteroides* 32–77:1, *Lactobacillus paracasei* subsp paracasei 19, and *Lactobacillus plantarum* 2362 and 2.5 g of each of the four fermentable fibres (prebiotics): betaglucan, inulin, pectin and resistant starch (Synbiotic AB, Höganäs, Sweden) [47,48].

Lund University microbiologists Åsa Ljungh and Torkel Wadström developed this multi-strain/multi-fibre synbiotic formula, which in recent years has been extensively used in clinical trials. The choice of LAB for the formulation was done after extensive studies of > 350 human [47] and >180 plant microbial strains [48] and based especially the ability of the LAB to produce bioactive proteins, transcribe NF-κB, produce pro- and anti-inflammatory cytokines, produce antioxidants, and most important, to functionally complement each other. In recent studies both the Synbiotic 2000 FORTE™ but also a Probiotic 2000 FORTE™ (no fibre added), containing 10^11^ of each of the four LAB, e.g. 400 billion LAB per dose have been tried.

### Plantarum, paracasei and pediococcus

When, as mentioned above, the ability of 712 different LAB to ferment oligofructans was studied, only 16/712 were able to ferment semi-resistant fibred; the phleins and 8/712 inulin; *Lactobacillus plantarum, Lactobacillus paracasei* subsp. p*aracaseiPediococcus pentosaceus* and *Lactobacillus brevis* and were able to ferment these semi-resistant fibres [15]. Interesting clinical results are also often obtained when these LAB are involved. When more than 100 LAB were compared *Lb paracasei* subsp *paracasei* were demonstrated to be the strongest inducer of Th1 & repressor of Th2 cytokines [49]. Several other studies have also documented the unique ability of *Lb paracasei* to iinduce cellular immunity, stimulate production of suppressive cytokines as TGFβ and Il-10, suppress CD4 T-cells, suppress Th2 activity, suppress splenocyte proliferation and decrease antigen-specific IgE and IgG1 [50-53].

The effect of *Lactobacillus paracasei* (NCC 2461), *Lactobacillus johnsonii* (NCC 533) and *Bifidobacterium lactis* Bb12 (NCC 362) on the induction and maintenance of oral tolerance to bovine beta-lactoglobulin (BLG) was investigated in mono-colonized germfree mice. The effects of *L. paracasei* were reported superior to those of the other two [53]. A study, which compared the ability of 50 different LAB to control 23 different pathogenic *Clostridium difficile* found more than half (27/50) totally ineffective, (18/50) antagonistic to some , but only five strains effective against all: two strains of *Lb paracasei* s. *paracasei* and five strains of *Lp plantarum*[54]. Another study compared the effects in rats receiving during days 10 to 21 after *Trichinella spiralis -* induced infection either *Lactobacillus paracaseiLactobacillus johnsoniiBifidobacterium longum*, or *Bifidobacterium lactis; Lb paracasei* but not the other LAB attenuated muscle hyper-contractility, reduced the infection-associated Th- 2 response and muscle levels of TGF-β, COX-2 and PGE2 [55]. A recent study compared in animals the effects of three probiotic strains: *Bifidobacterium lactis* NCC362, *Lactobacillus johnsonii* NCC533, and *Lactobacillus paracasei* NCC2461 on stress-induced changes in gut permeability & on sensitivity to colorectal distension (CRD) Only *Lb paracasei* both prevented reduced significantly existing visceral pain and also restored normal gut permeability [56].

### Synbiotic 2000 in clinical medicine

I have dedicated most of my clinical research during the last two decades to studies on effects of synbiotic compositions in various clinical situations, during the 1990s to the monostrain/monofibre composition, mentioned above, and 2000s to the multistrain/multifibre composition, also mentioned above; Synbiotic 2000 and 2000 Forte res. Here follows a summary of the effects observed this far. It has been tried in the following conditions:

#### Acute pancreatitis

**Figure 1:**
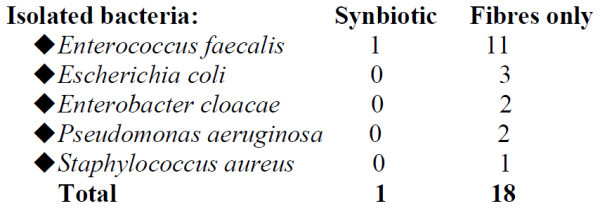
Isolated bacteria in acute pancreatitis treated with Synbiotic 2000 or Only the fibres.

Sixty-two patients with severe acute pancreatitis (SAP) (Apache II scores: Synbiotic 2000-treated 11.7 ± 1.9, controls 10.4 ± 1.5) were given either two sachets/day of Synbiotic 2000*™* (2x40 billion LAB/day and totally 20 g fibres) or the same amounts of fibres in (20 g) as in Synbiotic 2000*™* during the first 14 days after arrival to the hospital[57]. 9/33 patients (27%) in the Synbiotic 2000-treated group and 15/29 patients (52%) in the only fibre-treated group developed subsequent infections. 8/33 (24%) Synbiotic 2000-treated and 14/29 (48%) of the only fibre-treated patients developed SIRS, MOF or both (*p* < 0.005). A total of pathogenic microorganisms of seven were cultivated in the Synbiotic treated group compared to seventeen in the fibre-only group (Figure 1).

##### *Polytrauma*

Two prospective randomized trials one with Synbiotic 2000 and one with Synbiotic 2000 Forte respectively have been concluded. The first study compared in patients with acute extensive trauma: 1. Synbiotic 2000 (40 billion LAB/d) with 2. a soluble fibre 3. a peptide diet (Nutricomp, Braun Inc Germany) and 4. supplementation of glutamine. Treatment with Synbiotic 2000™ lead to a highly significant decrease in number of chest infections (4/26 patients - 15%), compared to peptide diet (11/26 patients - 42%, *p* < 0.04), glutamine (11/32 patients - 34%, *p* < 0.03) and only fibres (12/29 patients- 41%, *p* < 0.002) [58]. Also the total number of infections were significantly decreased; Synbiotic 2000™ 5/26 patients (19%) only fibres 17/29 patients (59%) peptide 13/26 patients (50%) and glutamine16/32 patients (50%).

**Figure 2:**
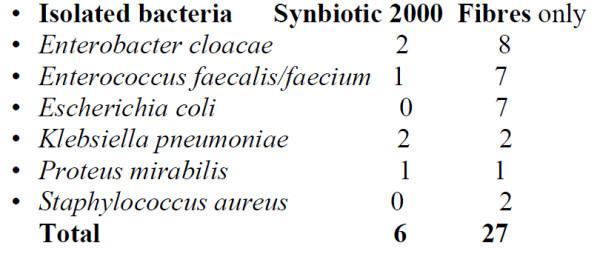
Isolated bacteria in patients undergoing pancreatoduodenectomy and treated with Synbiotic 2000 or Only the fibres.

In the second study sixty-five patients polytrauma patients were randomized to receive once daily for 15 d Synbiotic 2000 Forte (400 billion LAB + 10 gram of fibres, see above) or maltodextrine as placebo. Significant reductions were observed in number of deaths (5/35 vs 9/30, *p* < 0,02), severe sepsis (5/35 vs 13/30, *p* < 0.02), chest infections (19/35 vs 24/30, *p* < 0.03), central line infections (13/32 vs 20/30, *p* < 0.02), and ventilation days (average 15 vs 26 days) [59]. A total of pathogenic microorganisms of 54 were cultivated in the Synbiotic treated group compared to 103 in the placebo group. The time to progression to primary bacteraemia was longer among patients treated with Synbiotic 2000 Forte compared with placebo (*p* _ 0.0237 between groups). Twelve (33.3%) and five (13.9%) placebo-treated and synbiotic-treated patients, respectively, developed ventilator-associated pneumonia with *Acinetobacter baumannii* as a bacterial cause (*p* 0.047 between groups). Treatment with Synbiotic 2000 Forte was accompanied by reduction of white blood cell counts and LPS and CRP levels in either patients who did or did not develop sepsis [60]. *Abdominal surgery:* In a randomized controlled study forty-five patients undergoing major surgery for abdominal cancer were divided into three treatment groups: 1. enteral nutrition (EN) + Synbiotic 2000 (LEN), 2. EN + only the fibres in the same amounts (20 g) (20 g) as in Synbiotic 2000*™* (FEN) and 3. A standard parenteral nutrition (PN). All treatments lasted for 2 preoperative and 7 days postoperative days. The incidence of postoperative bacterial infections was 47% with PN, 20% with FEN and 6.7% with LEN (*p* < 0.05) (Han Chun Mao et al in press). A total of pathogenic microorganisms of 34 were cultivated in the Synbiotic treated group compared to 54 in the fibre-only group. Significant improvements were also documented in prealbumin (LEN, FEN), C-reactive protein (LEN, FEN), serum cholesterol (LEN,FEN), white cell blood count (LEN) , serum endotoxin (LEN, FEN) and IgA (LEN). In another prospective randomized double-blind trial performed in 80 patients subjected to pylorus-preserving pancreatoduodenectomy (PPPD) receive twice daily either Synbiotic 2000^TM^ (2x40 billion LAB) or only the fibres in composition from the day before surgery and during the first seven postoperative days [61]. A highly significant difference in infection rate (*p* = 0,005) was observed as only 5/40 patients (12.5%) in the Synbiotic 2000-treated group suffered infections (4 wound and one urinary tract infection) versus 16/40 (40%) in the Only fibre group (6 wound infections, 5 peritonitis, 4 chest infections, 2 sepsis, and one of each of urinary tract infection, cholangitis and empyema). The infecting microorganism were in the Synbiotic treated group *Klebsiella pneumoniae* (2 patients), *Enterobacter cloacae* (2 patients), *Proteus mirabilis* (1 patient) and *Enterococcus faecalis/faecium* (1 patient) and in the Only fibre group *Enterobacter cloacae* (8 patients), *Enterococcus faecalis/faecium* (7 patient), *Escherichia coli* (7 patient), *Klebsiella pneumoniae* (2 patients), *Staphylococcus aureus* (2 patients), and *Proteus mirabilis* (1 patient) - see further Figure 2. Statistically significant differences between the groups were also observed in use of antibiotics (mean: Synbiotic 2000; 2 ± 5 days, Only fibres; 10 ± 14 days).

##### *Chronic liver disease and liver transplantation*

Fifty-eight patients with liver cirrhosis suffering so called minimal encephalopathy were randomized into three treatment groups: Group 1 (20 patients) received Synbiotic 2000 (40 billion LAB), group 2. (20 patients) received the same amount of the fibres in Synbiotic 2000 and group 3. (15 patients) received placebo (non-fermentable, non-absorbable fibre -crystalline cellulose) [62]. A significant increase in intestinal LAB flora was observed after one month of supplementation in the Synbiotic-treated group, but not in the other two groups. Intestinal pH was significantly reduced in both treatment groups but not in the placebo-treated group. Significant decreases in faecal counts of *Escherichia coli, Staphylococcus* and *Fusobacterium,* but not in *Pseudomonas* and *Enterococcus,* and significant decreases in ammonia/s, endotoxin/s ALT/s and bilirubin/s (original level 252 ± 182) were observed in the Synbiotic 2000-treated group ( 84 ± 65, *p* < 0.01) and in the Only fibre-treated group (110 ± 86, *p* < 0.05) while it remained unchanged in the placebo group. The improvements in liver function were accompanied by significant improvements in psychometric tests and in degree of encephalopathy.

In a follow-up study by the same group of investigators 30 patients with liver cirrhosis were randomized to receive either Synbiotic 2000 or placebo (crystalline cellulose ) for 7 days [63]. Viable fecal counts of *Lactobacillus species*, Child-Pugh class, plasma retention rate of indocyanine green (ICG_R15_), whole blood tumour necrosis factor alpha (TNF--6 (IL-6) mRNA, serum TNF-α, soluble TNFreceptor (sTNFR)I, sTNFRII and IL-6 and plasma endotoxin levels were measured pre- and post-treatment: Synbiotic treatment was associated with significantly increased faecal lactobacilli counts and significant improvements in plasma retention rate of indocyanine green (ICG_R15)_ and stage of liver disease (Child-Pugh classification). No significant changes in any study parameter followed placebo treatment, but significant increases in whole blood whole blood tumour necrosis factor alpha (TNF- α) mRNA and interleukin-6 (IL-6) mRNA, along with serum levels of soluble TNF receptors sTNFRI and sTNFRII, were observed in the Synbiotic 2000-treated patients. TNF- α and IL-6 levels correlated significantly, both at baseline and post-synbiotic treatment. Synbiotic-related improvement in ICG_R15_ was significantly associated with changes in IL-6, both at mRNA and protein levels, and unrelated to plasma endotoxin values. It was concluded that even short-term synbiotic treatment can significantly modulates gut flora and improve liver function in patients with cirrhosis. The observed benefits seemed unrelated to reduction in endo-toxaemia, but could be mediated, at least in part, by treatment-related induction of IL-6 synthesis by TNF- α. These results offers great hope that synbiotic treatment to patients on waiting list for liver transplantation might prevent septic episodes, improve liver function, and promote successful outcome of surgery.

**Figure 3:**
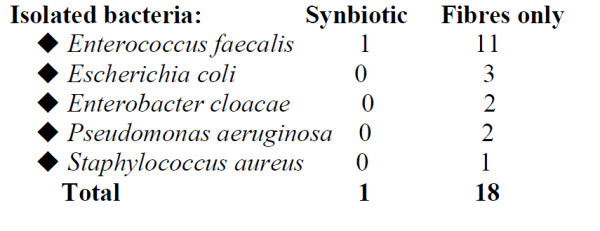
Isolated bacteria in patients undergoing liver transplantation and treated with Synbiotic 2000 or Only the fibres.

Sixty-six patients were randomized to either receive Synbiotic 2000 or only the fibres in Synbiotic 2000 in connection with human ortotopic liver transplantation. The treatment started already on the day before surgery and continued for 14 days after surgery. During the first postoperative month did only one patient in the Synbiotic 2000-treated group (3%) show signs of infection (urinary infection) compared to 17/33 (51%) in the patients supplemented with only the four fibers [64]. The infecting organisms were in the Synbiotic-treated group *Enterococcus fecalis* in 1 patient and in the only fibre-treated group *Enterococcus fecalis/fecium* 11, *Escherichia coli* 3, *Enterobacter cloacae* 2, *Pseudomonas aeruginosa* 2 and *Staphylococcus aureus* in 1 patient, see further Figure 3. The use of antibiotics was in average 0.1 ± 0.1 d in the synbiotic-treated patients and 3.8 ± 0.9 d in the only fibre-treated group.

##### *Inflammatory bowel disease*

Daily rectal instillations with Synbiotic 2000 reconstituted in saline were given to ten patients with distal colitis during 2 weeks. One patient withdraw after one week, the remaining patients showed during the 3 weeks of observation dramatic improvements in various disease scores; episodes of diarrhoea ((2.4 ⇒ 0.8), visible blood in stool (2.2 ⇒ 0.8), nightly diarrhoea (0.5 ⇒ 0), urgency (1.9 ⇒ 1.0) and consistency of stool ((1.1 ⇒ 0.8) [65]. Two patients reported significant bloating and wind but no other adverse or side effects were reported.

### Treatment-resistant conditions

Treatment with synbiotics have not this far improved the disease in two groups of conditions:

*Inflammatory bowel disease - Crohn’s Disease (CD):* Sixty-three patients were after an initial treatment with infliximab randomized to daily receive either Synbiotic 2000 or crystalline cellulose as placebo [66]. Median time to relapse was 9.8 and 10.1 months respectively. In a second study were following surgery patients supplemented with either Synbiotic 2000 or crystalline cellulose as placebo. Seven patients in the synbiotic-treated group and two in the placebo group completed the scheduled 24 month treatment [67]. No differences were observed between the two groups either in endoscopic findings or rate of clinical relapse. The so called Rutgeerts disease scores were after three months of treatment 0.6 ± 0.8 in the synbiotic-treated group and 0.8 ± 1 in the placebo group (NS).

*General intensive care patients:* Two large studies have been performed in a general intensive care population; one with Synbiotic 2000 and one with Synbiotic 2000 FORTE. Synbiotic 2000 (40 billion LAB) was given to 162 patients and only fibre the fibres in the synbiotic composition to 168 patients. No difference was observed in mortality or in multi-organ dysfunction [68]. In another study Synbiotic 2000 FORTE 130 patients were supplemented twice a day throughout the whole ICU stay to (2 × 400 billion LAB) and compared to 129 patients supplemented a cellulose based placebo. No statistical difference was demonstrated between the groups in the incidence of VAP (9 and 13%, *P* = 0.31). The rate of ventilator-associated pneumonia (VAP) per 1000 ventilator days was 13 and 14.6 (*p* = 0.73) and hospital mortality 27 and 33%, (*p* = 0.32), respectively [69].

## Conclusions

Thousands of factors are important to maintain health and to cure disease. This might explain why single drug pharmacy fails, both to prevent disease and to cure disease especially when chronic. Human innate immunity is for a proper function much depending on continuous access to bacteria and plants. Using probiotics in combination with plants and their active ingredients remains an attractive approach for prevention and treatment of various both acute and chronic diseases. Ten year old studies in the United States demonstrate an 83 percent reduction in rate of coronary heart disease [70], a 91 percent reduction in diabetes in women [71], and a 71 percent reduction in colon cancer in men [72] in patients adhering to what is regarded as an “healthy lifestyle”: no use of tobacco, moderate use of alcohol, regular physical exercise, and eating a diet low in animal fat, low in refined carbohydrates, and rich in fresh fruits and vegetables (if raw also rich in lactic acid bacteria) and fish, a diet often referred to as Mediterranean diet.

Crohn’s disease, as an example, has despite vigorous attempts over the years remained most resistant to therapy, pharmaceutical as well as probiotic treatments -see recent review [73]. Solid observations suggest that several Th1-mediated autoimmune diseases, such as multiple sclerosis, type 1 diabetes, rheumatoid arthritis and Crohn´s disease are associated with low vitamin D status, and a recent study demonstrates strong molecular links between vitamin D deficiency and the genetics of Crohn’s disease [74]. Crohn’s disease is also strongly associated with lack of minerals, especially calcium and magnesium. Hypomagnesaemia is known to be strongly associated with increased systemic inflammation, manifesting in leukocyte and macrophage activation and increased production of inflammatory cytokines and acute phase proteins. A recent study demonstrated a much deranged microbionta in experimental animals with induced hypomagnesemia [75]. It is certainly too much to request that pre-, pro-, or synbiotics shall make a difference in such conditions unless the underlying defects in metabolism and immune functions are corrected.

Inflammation is “the mother of disease” and much associated with the food we eat [76,77]. As pointed out above, numerous factors contribute to the deranged innate immune system and the sustained exaggerated inflammation. Numerous changes are also required to permanently control the hyper-inflammation and subsequent disease. Supply of pre-, pre-, and synbiotics are strong tools for such corrections of immune functions and resistance to disease. However, in most instances also other measures are necessary, including reduction in intake of pro-inflammatory molecules [2,78], and also substantial intake of anti-inflammatory food ingredients such as turmeric/curcumin [79,80].

The critical care units constitute a highly artificial environment and the burden of environment-induced physical and mental stress and subsequent status of systemic hyper-inflammation on the patient is unbearable. Patients treated under these conditions are in many aspects dys-functional, the whole microbiota is more or less gone and probiotic bacteria supplied will most often be killed already before they have reached the lower gastro-intestinal tract. The degree of artificiality seems to vary from hospital to hospital and from country to country, which might explain the great variation in outcome from studies undertaken in different regions and countries.

Probiotic treatment has never been given the chance as an alternative treatment, it has only this far been tried as a treatment complementary to all the other standard treatments. As discussed above, a series of auxiliary measures and particularly mechanical ventilation and treatment with various drugs, including antibiotics but also clinical nutrition solutions belong to those factors which promote super-inflammation and indirectly infection. Enteral nutrition formulas are also known to induce loss of intestinal barrier function, promotes bacterial translocation, and impair host immune defense, a phenomenon, observed in humans and further elucidated in animal studies.

## Competing interests

I am shareholder in various probiotic companies.
